# Differences in age-specific HPV prevalence between self-collected and health personnel collected specimen in a cross-sectional study in Ghana

**DOI:** 10.1186/s13027-017-0136-7

**Published:** 2017-05-18

**Authors:** Adolf K. Awua, Richard M. K. Adanu, Edwin K. Wiredu, Edwin A. Afari, Alberto Severini

**Affiliations:** 1 Department of Epidemiology and Disease Control, School of Public Health, College of Health Sciences, University of Ghana, Accra, Ghana; 2 Cellular and Clinical Research Centre, Radiological and Medical Sciences Research Institute, GAEC, Accra, Ghana; 3 Population, Family and Reproductive Health, School of Public Health, College of Health Sciences, University of Ghana, Accra, Ghana; 4 Department of Pathology, School of Biomedical and Allied Health Science, College of Health Sciences, University of Ghana, Korle-Bu, Accra, Ghana; 5 National Microbiology Laboratory, Public Health Agency of Canada, Winnipeg, MB Canada; 6 University of Manitoba, Winnipeg, MB Canada

**Keywords:** Human Papillomavirus, Age-specific, Cervical cancer, Self-sample collection, Screening, Ghana

## Abstract

**Background:**

HPV infections are ubiquitous and particularly common among sexually active young women. However, there are regional and national variations in age-specific HPV prevalence, which have implications for cervical cancer control. Data on age-specific HPV prevalences for Ghana and most sub-Saharan countries are scanty. Therefore, this study primarily sought to determine the age-specific HPV prevalence among women in a Ghanaian community and to determine whether these prevalences determined with health-personnel and self-collected specimens were comparable.

**Methods:**

In this cross-sectional study, conducted between March 2012 and March 2013, cervical specimens were collected by self- and health-personnel collection from 251 women who were between the ages of 15 and 65 years. HPV present in these specimens were genotyped by a nested-multiplex PCR and Luminex fluoro-microspheres based method. Information on the demographic, sexual and reproductive characteristics of the women were also obtained. A Chi-square test of association was employed to determine the association of the distribution of age groups with each categorised sexual and reproductive characteristic and HPV risk type’s status.

**Results:**

The age group distribution of the participants was significantly associated with overall (*χ* ^2^ = 36.1; *p* = 0.001), high risk (*χ* ^2^ = 26.09; *p* = 0.002) and low risk (*χ* ^2^ = 21.49; *p* = 0.011) HPV prevalences. The age-specific HPV prevalence pattern for each of the HPV risk types, determined with self-collected specimen, showed three peaks (at 20–24 years; 40–44 years and ≥ 55 years), while those determined with health-personnel collected specimen, showed two peaks (at 20–24 years and ≥ 55 years) for each HPV risk type’s prevalence pattern. The high risk HPV prevalences determined with self-collected specimen were often higher than those determined with health-personnel specimen for the age groups between 25 and 45 years, who are mostly targeted for screening by HPV testing. Additionally, there were interesting variations in patterns of age-specific HPV genotype-specific prevalence between the two specimen collection methods.

**Conclusions:**

The usefulness of self-collected specimen for high risk HPV burden determination and the existence of a two peaked and three peaked age-specific HPV prevalences in Ghana have been clearly indicated.

**Electronic supplementary material:**

The online version of this article (doi:10.1186/s13027-017-0136-7) contains supplementary material, which is available to authorized users.

## Background

Human Papillomaviruses are small, single coated, double stranded DNA virus that have been classified to belong to 30 species and about 200 distinct genotypes [1–3]. Of these, about 50 types infect the mucosal epithelia of most parts of the human body, including the cervix, vagina, vulva, penis, anus and the oropharynx [1, 3]. They are transmitted by almost all forms of sexual contacts, including penetrative intercourse, oral sex, genital contacts and genital skin to skin contact as well as by self-inoculation [4, 5]. These ubiquitous viruses are particularly common among young sexually active women; however, variations in the age specific cervical HPV prevalence, within and between countries and the WHO world regions have been well documented [6]. Similar variations have been reported for other genital tissues, oral, head and neck malignancies [7].

Based on a meta-analysis of 78 studies, which used health personnel collected specimen reported and data of women with normal cytology [6], and also on a few research studies (which used self-collected specimen [8]) and other articles, which did not indicate specimen collection [9, 10], it was clear that women who were younger than 25 years were the most infected by HPV (with a peak between 20 and 22 years). This peak was followed by a progressive decrease in prevalence as age increased until a small increase was observed among women 45–54 years from Africa and Europe, and between 34 and 45 years for those from the North, Central and South America regions. Data from the Asia region did not show a second increase in prevalence as observed for the other regions [6]. Further to these, pooled analysis of country specific multi-centre study HPV data, obtained with health personnel collected specimen from women with normal cytology, showed only a single peak at 25–34 years [11], although data from the individual countries showed different prevalence trends. For instance, data from the Nigerian study in this pooled data showed two peaks, at 25–34 years and 45–54 years; while data form Italy and Netherlands showed one peak, at 55–64 years and 45–54 years respectively; data from Thailand and Vietnam showed two peaks. at 25–34 years and 55–64 years; data form Columbia, Argentina and Chile all showed one peak, but at 25–34 years, 35–44 years and 35–44 years respectively [11]. Similarly a Canadian study, which tested for only high risk HPV types using health-personnel collected specimen, showed a single peak at 20–24 years [12], while as reported by Bosch et. al., [13] a study in Spain, that used health personnel collected specimen, showed two peaks at 20–24 years and 45–54 among the general population of women.

For reasons not clearly understood, the variation in age-specific HPV prevalence trends for African countries seems to have both one peak (unimodal) and two peaks (bimodal) but not three peaks (trimodal) prevalence. That is, in rural Nigeria, South Africa and Senegal an initial high peak among younger women was followed by a second small prevalence peak among mid-age women (bimodal prevalence) [14–17]. In other studies conducted in South Africa, Nigeria and Mozambique, it was determined that HPV prevalence reduced with increase in age, showing a peak (unimodal) among younger women [14, 18–20]. In the Gambia, the age-specific HPV prevalence was seemingly consistent with increase in age [21]. It is worth noting that these and most age-specific prevalence studies had been based on the analysis of specimen collected by health personnel (HPC), particularly for Pap testing [6].

An effective cervical cancer screening strategy, referred to as self-collection has gained a lot research attention in recent times [22–24]. This strategy involves women themselves collecting cervicovaginal swabs in the privacy of their home for HPV testing, and has been compared with cytology and HPC-HPV testing for the reduction of cervical intraepithelial neoplasia (CIN) and the determination of overall HPV prevalence. Such studies reported a variety of Kappa estimates (ranging between 0.24 and 0.96) and concordances (ranging between 70 and > 90%) for the overall HPV prevalence agreement between self-collected (SC) and HPC specimen [22, 23, 25–29]. Most of these studies have suggested that the difference in overall HPV prevalence between these methods may be due to LR HPV preferentially infecting the vagina than the cervix and therefore they become more detectable with SC than HPC, since the time-to-clearance were similar for both LR HPV and HR HPV detected by both methods [22, 23, 25–28].

Among these studies, only the studies from South Africa [26] and Uganda [27] presented a limited comparison of the age-specific HPV prevalences obtained with the two collection methods, and these showed a non-significant difference. Therefore, the aim of our study was to determine the differences, if any, between the age-specific HPV prevalences determined with SC specimen and those determined with HPC specimen in Ghana and compare the age-specific HPV detection concordance between the two methods. We present the first age-specific HPV prevalence for a Ghanaian population and an assessment of the distribution of related participant characteristics stratified by age.

## Methods

### Study population

In this cross-sectional study of the multi-ethnic population of the Akuse sub-district, women were recruited between March 2012 and March 2013 by a house survey and invited for specimen collection either at the Akuse Government Hospital or at a specified location within the individual communities. In each of 17 communities, the major roads/paths through it were identified by the use of Google Map computer application and grouped in order of direction, from one end of the community to the other. Based on the crude estimate of the average number of houses and the population of women in each of the communities, it was assessed that visiting every 5th house or less will ensure a wide coverage of the communities. Therefore, starting from the first randomly selected road/path, every third house to the right and left of the road/path was visited. Not more than three women in each house, who met the inclusion criteria were invited to participate in the study, after explaining the study, the consent form and the options available to them as participants as well as giving them basic information about cervical cancer.

Women between the ages of 15 and 65 years who had ever been sexually active and were willing to provide specimen by at least one of the two specimen collection methods (data not shown) were included in this study. Women having their menses were asked to come back 1 week after their menstrual period. Women who were pregnant (self-report), had undergone hysterectomy or cervical conisation were excluded from the study. Two trained Public Health Nurses assisted the study participants, by a one-on-one interview, to complete a study questionnaire that obtained information on the socio-demographic characteristics, sexual behaviour, sexual and reproductive history, menstrual factors, use of oral contraception and history of sexually transmitted infections and cervical cancer screening history.

### Compliance with ethical standards

The study was conducted in accordance with the ethical standards as laid down in the 1964 Declaration of Helsinki and its later amendments and comparable ethical standards. The participants either read and signed an informed consent form (in English) or it was translated to a local language and explained to them with the assistance of a witness; the explanation in each of the four local languages (Hausa, Ewe, Ga-Adangbe and Twi) was performed by one particular person throughout the study. The informed consent included the main research goals, sample collection procedures, potential benefits and harms and privacy and confidentiality. The name and mobile phone number (s) (personal or that of another family member) of the women were obtained and each was recorded at separate places alongside a unique identification code, after informed consent had been obtained. The study was approved as a part of a bigger study by the Ethics Review Committee of the Ghana Health Service (ID No GHS-ERC: 06/11/10).

### Specimen collection and smear preparation

Each participant was instructed on how to use the Rover® Viba-Brush vaginal sampler, (Rovers Medical Devices, The Netherlands). The instructions were given verbally and in the language the participant understood (the option of four local languages was available) with demonstrations, using an illustrations on the packaging of the brush, that showed the steps and positioning of the body. The participants were asked to describe the process they going to following to the investigator, before they allowed to go to the private room to perform the specimen collection. The collected specimen was transferred into a sterile 15 mL screw-capped tube containing 5 mL PBS with 5 mM ethylene diamine tetraacetic acid (EDTA), pH 8.0. These specimens were designated as self-collected (SC) for HPV testing.

The health personnel then performed a standardized pelvic examination and with the aid of a sterile plastic disposable speculum, a wooden spatula and a cytobrush collected cervical specimen according to the manufacturer’s instructions. The cytobrush was then rinsed in 50 μL of DNAgard®Tissue (Biomatrica, Inc., San Diego) and head of the wooden spatula was rinsed in the same 50 μL of DNAgard®Tissue. The specimen was designated as Health Personnel Collected (HPC). Both collected specimens were transported at 4 °C and thereafter store at −20 °C until used for HPV testing.

### DNA extraction

The volume of specimen obtained by HPC was made-up to 1 mL with PBS and vortexed thoroughly. DNA was extracted from 200 μL of each specimen (SC and HPC) on the MagNA Pure LC automated system (Roche Molecular Systems, Inc, Pleasanton, USA) using the MagNA Pure DNA Extraction Kit (Roche Molecular Systems, Inc, Pleasanton, USA) according to the manufactures specification and a modification by adding 15 μL of RNase to the lysis buffer for each specimen. The negative controls included for each set of extraction were PBS, DNase/RNase free water and DNAGard while the positive control was a suspension of SiHa cells with an integrated HPV 16 genome. The specimen DNA quality was assessed by a real-time PCR amplification of the house keeping gene, RNase H in 10 μL of DNA extract and analysed with Light Cycler 480 and related software (Roche Molecular Systems, Inc, Pleasanton, USA).

### Nested-multiplex PCR based detection of HPV genotype

The extracted DNA was amplified by a nested PCR reaction using PGMY09/PGMY11 and GP5+/GP6+ primers as previously described [30].

The typing of 46 mucosal HPV types was carried out by a multiplex system based on the xMAP® technology as previously described by Zubach et al., [30]. Briefly, 46 fluorescence sortable microspheres (Luminex Corporation, Austin, TX) were coupled to the 46 specific probes for HPV types 6, 11, 13, 16, 18, 26, 30, 31, 32, 33, 34, 35, 39, 40, 42, 43, 44, 45, 51, 52, 53, 54, 56, 58, 59, 61, 62, 66, 67, 68, 69, 70, 71, 72, 73, 74, 81, 82, 83, 84, 85, 86, 87, 89, 90 and 91.. The double stranded second round PCR products, labelled with biotin, were made single-stranded by digestion with 2 μL of bacteriophage T7 gene6 exonuclease (New England BioLabs, Pickering, ON, Canada) that removed the non-labelled strand after 40 min incubation at room temperature. The single stranded HPV DNA was incubated for hybridization at 60 °C for 10 min and streptavidin-phycoerythrin (PE) (Invitrogen) in 1-tetramethyl ammonium chloride (TMAC) (Sigma), was added and incubated for 5 min at 60 °C. Genotype specific hybridization was detected on a Luminex Liquid Chip 200 flow cytometer (Qiagen) using the Luminex IS software (Luminex).

### Statistical analysis

All the participants’ sexual and reproductive characteristics were categorised into the known groups widely reported for HPV risk factors analysis. The age of the participants were also categorised into groups of 5 year intervals. Frequencies and proportions were used to describe the distributions and summarize data of demographic characteristics and the categorized sexual and reproductive characteristics stratified by age groups (5 year intervals). Chi-square test of association (at 95% confidence level) was used for the determination of the univariate association between the categorised participants’ characteristics and grouped age, and between grouped age and the HPV prevalence for each of the HPV risk types and for overall HPV. A bivariate and multivariate Chi-square association were subsequently determined for each of the HPV risk types and for overall HPV For the Chi-square analysis of the age at first sexual intercourse (AFSI) stratified by age group, the analysis was conducted, first, excluding women 20 years and younger and secondly, grouping the women within the age groups 15–19 and 20–24 to a new group labelled as < 25 years. For both analyses the association was significant by Chi-square test at 95% confidence level. The Cohen’s Kappa analysis was used to determine the extent of agreement between the two methods for the detection of the following; overall/any HPV type, high risk types and low risk types, using data of only 226 women who provided both specimen. For overall HPV type, agreement was defined as Individuals positive for any HPV genotypes in both samples. For high risk HPV, agreement was defined as Individuals positive for any of the high risk HPV genotypes in both. For the Low risk HPV, agreement was defined as Individuals positive for any of the Low risk HPV types in both samples. For of the three criteria it was not necessarily the same genotype in both samples.

## Results

### Specimens

Of the 415 women contacted during the study, 258 women provided specimens. Of this number 4 self-collected (SC) and 3 health personnel collected (HPC) specimens were excluded because their labels were destroyed during handling of the samples. The quality of the specimens of the remaining 251 women was found to be appropriate for further analysis; although 17 of the 244 SC and 6 of the 233 HPC specimen were not consistently positive with triplicate analyses for the RNase H gene (2 of 3 were positive); these specimen were included in the HPV analysis. Overall 226 women provided both SC and HPC specimen. The distribution of the demographic characteristics of the 415 women and those of them who were HPV positive are shown in Table 1.

**Table 1:** Distribution of the demographic data of the participants who were positive for HPV by at least one of the specimen collected

Demographic characteristics	Sub-categories	Frequency (%)	
		All Women	Women HPV positive by any specimen
Grouped Age	15–19	26 (6.3)	8 (6.5)
	20–24	65 (15.8)	28 (22.6)
	25–29	97 (23.5)	34 (27.4)
	30–34	46 (11.2)	8 (6.5)
	35–39	52 (12.6)	13 (10.5)
	40–44	51 (12.4)	15 (12.1)
	45–49	30 (7.3)	3 (2.4)
	50–54	22 (5.3)	6 (4.8)
	55–59	13 (3.2)	8 (6.5)
	60 Or Older	10 (2.4)	1 (.8)
	Total	412 (100.0)	124 (100.0)
Religion	Christian	373 (90.5)	121 (97.6)
	Muslim	35 (8.5)	3 (2.4)
	Other	4 (1.0)	0.0 (0.0)
	Total	412 (100.0)	124 (100.0)
Marital Status	Unmarried	150 (36.9)	47 (37.9)
	Married	257 (63.1)	74 (59.7)
	Total	407 (100.0)	124 (100.0)
Educational Status	No Formal	68 (16.6)	19 (15.3)
	Primary	77 (18.8)	25 (20.2)
	Junior Secondary	194 (47.4)	56 (45.2)
	Senior Secondary	48 (11.7)	11 (8.9)
	Post-Secondary	22 (5.4)	13 (10.5)
	Total	409 (100.0)	124 (100.0)
Occupation	Unemployed	25 (6.2)	9 (7.3)
	Formally employed	66 (16.4)	25 (20.2)
	Skilled Worker	93 (23.1)	34 (27.4)
	Trader	172 (42.8)	43 (34.7)
	Agro-worker	46 (11.4)	8 (6.5)
	Total	402 (100.0)	124 (100.0)

### Age specific distribution of characteristics

Following the stratification of the distribution of the sexual and reproductive characteristics by age (Table 2), it was determined that a significant generational difference in age at first sexual intercourse (AFSI) existed for this population (*χ* ^2^ = 42.8, *p* = 0.0001). Also, the distributions of lifetime number of male sexual partners (*χ* ^2^ = 34.6, *p* = 0.01), the contraction of a sexually transmitted infection (STI) (*χ* ^2^ = 44.4, *p* = 0.0001), use of condoms (*χ* ^2^ = 52.0, *p* = 0.0001), current number of male sexual partners (*χ* ^2^ = 72.3, 0.0001), number of pregnancies (*χ* ^2^ = 146.8, *p* = 0.001) and ever had an abortion (*χ* ^2^ = 29.1, *p* = 0.001) were all significantly associated with age. However, the use of OC was not significantly associated with age (*χ* ^2^ = 9.0, *p* = 0.437).

**Table 2:** Distribution and association of sexual and reproductive characteristics stratified by age

Risk factors	Category	Age group (years), n (%)									*χ* ^2^ (*p*-value)
		15–19^e^ (*n* = 26)	20–24^e^ (*n* = 64)	25–29 (*n* = 97)	30–34 (*n* = 47)	35–39 (*n* = 52)	40–44 (*n* = 51)	45–49 (*n* = 31)	50–54 (*n* = 24)	≥55 (*n* = 23)	
Age at first sexual intercourse	<20	16 (100)	56 (87.5)	53 (55.2)	25 (54.3)	30 (57.7)	34 (69.4)	16 (53.3)	9 (42.9)	11 (50.0)	42.8^e^ (0.0001)^a^
	≥20	0 (0.0)	8 (12.5)	43 (44.8)	21 (45.7)	22 (42.3)	15 (30.6)	14 (46.7)	12 (57.1)	11 (50.9)	
Lifetime number of sexual partners	1	8 (53.3)	19 (29.7)	22 (22.9)	5 (10.9)	9 (17.6)	11 (22.4)	7 (23.3)	4 (18.2)	6 (26.1)	34.6 (0.010)^a^
	2	5 (33.3)	21 (32.8)	30 (31.3)	16 (34.8)	12 (23.5)	13 (26.5)	6 (20.0)	4 (18.2)	4 (17.4)	
	≥3	2 (13.3)	24 (37.5)	44 (45.8)	25 (54.3)	30 (58.8)	25 (51.0)	17 (56.7)	14 (63.6)	13 (56.5)	
^c^Current number of male sexual partners	0	16 (61.5)	6 (9.4)	6 (6.2)	5 (10.9)	4 (7.7)	6 (12.0)	4 (13.3)	6 (27.3)	8 (36.4)	72.3 (0.0001) ^a^
	1	10 (38.5)	58 (90.6)	89 (91.8)	41 (89.1)	48 (92.3)	44 (88.0)	26 (86.7)	16 (72.7)	14 (63.6)	
Contracted a sexually transmitted infection in the past 10 years	No	10 (38.5)	18 (28.6)	36 (37.1)	18 (40.0)	22 (43.1)	34 (68.0)	15 (51.7)	16 (72.7)	20 (86.9)	44.4 (0.0001) ^a^
	Yes	16 (61.5)	45 (71.4)	61 (62.9)	27 (60.0)	29 (56.9)	16 (32.0)	14 (48.3)	6 (27.3)	3 (13.0)	
^b^Use of condom during sexual intercourse	No	12 (48.0)	28 (44.4)	38 (39.2)	24 (52.2)	36 (70.6)	40 (78.4)	22 (73.3)	19 (86.4)	20 (76.9)	52.0 (0.0001)^a^
	Yes	13 (52.0)	35 (55.6)	59 (60.8)	22 (47.8)	15 (29.4)	11 (21.6)	8 (26.7)	3 (13.6)	3 (23.1)	
Number of pregnancies	1	4 (80.0)	23 (43.4)	17 (21.5)	6 (13.3)	2 (4.1)	3 (6.0)	1 (3.4)	0 (0.0)	1 (4.5)	146.8 (0.0001)^a^
	2–4	1 (20.0)	27 (50.9)	54 (68.4)	27 (60.0)	17 (34.7)	17 (34.0)	10 (34.5)	4 (18.2)	5 (22.7)	
	≥5	0 (0.0)	3 (5.7)	8 (10.1)	12 (26.7)	30 (61.2)	30 (60.0)	18 (62.1)	18 (81.8)	16 (72.7)	
^d^Have you ever had any abortion	No	24 (92.3)	43 (68.3)	59 (60.8)	21 (45.7)	24 (46.2)	24 (47.1)	17 (56.7)	9 (40.9)	17 (73.9)	29.1 (0.001)^a^
	Yes	2 (7.7)	20 (31.7)	38 (39.2)	25 (54.3)	28 (53.8)	27 (52.9)	13 (43.3)	13 (59.1)	6 (26.1)	
Number of abortion	1	2 (100)	15 (75.0)	24 (63.2)	14 (56.0)	12 (42.9)	13 (48.1)	8 (61.5)	8 (61.5)	4 (66.7)	11.0 (0.272)
	≥2	0 (0.0)	5 (25.0)	14 (36.8)	11 (44.0)	16 (57.1)	14 (51.9)	5 (38.5)	5 (38.5)	2 (33.3)	
Use of oral contraceptive in the past 10 years	No	24 (96.0)	49 (77.8)	74 (77.1)	30 (65.2)	37 (75.5)	39 (78.0)	22 (75.9)	16 (72.7)	18 (78.3)	9.0 (0.437)
	Yes	1 (4.0)	14 (22.2)	22 (22.9)	16 (34.8)	12 (24.5)	11 (22.0)	7 (24.1)	6 (27.3)	5 (21.7)	

^a^Significant differences within age groups

^b^condom used was not consistent among all the participants

^c^only 2 women in age group 25–29 years had more than 1 current male sexual partner

^d^both spontaneous and induced abortion

^e^For the Chi-square analysis of AFSI stratified by age group, the analysis was conducted, first, excluding women 20 years and younger (the p value is reported) and secondly, grouping the women within the age group 15–19 and 20–24 to a new group labelled as < 25 years. For both analyses the association was significant by Chi-square test at 95% confidence level

### Age specific HPV prevalences

**Fig. 1:**
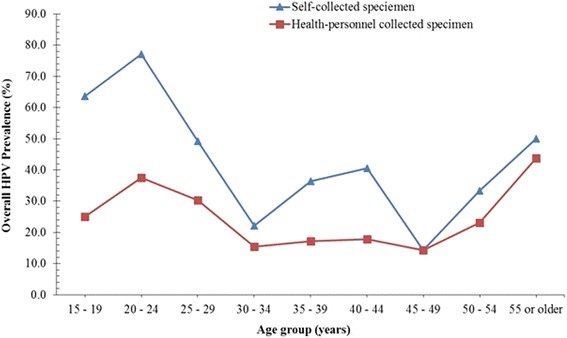
Overall HPV prevalence curves obtained with self and health personnel collected specimen

**Fig. 2:**
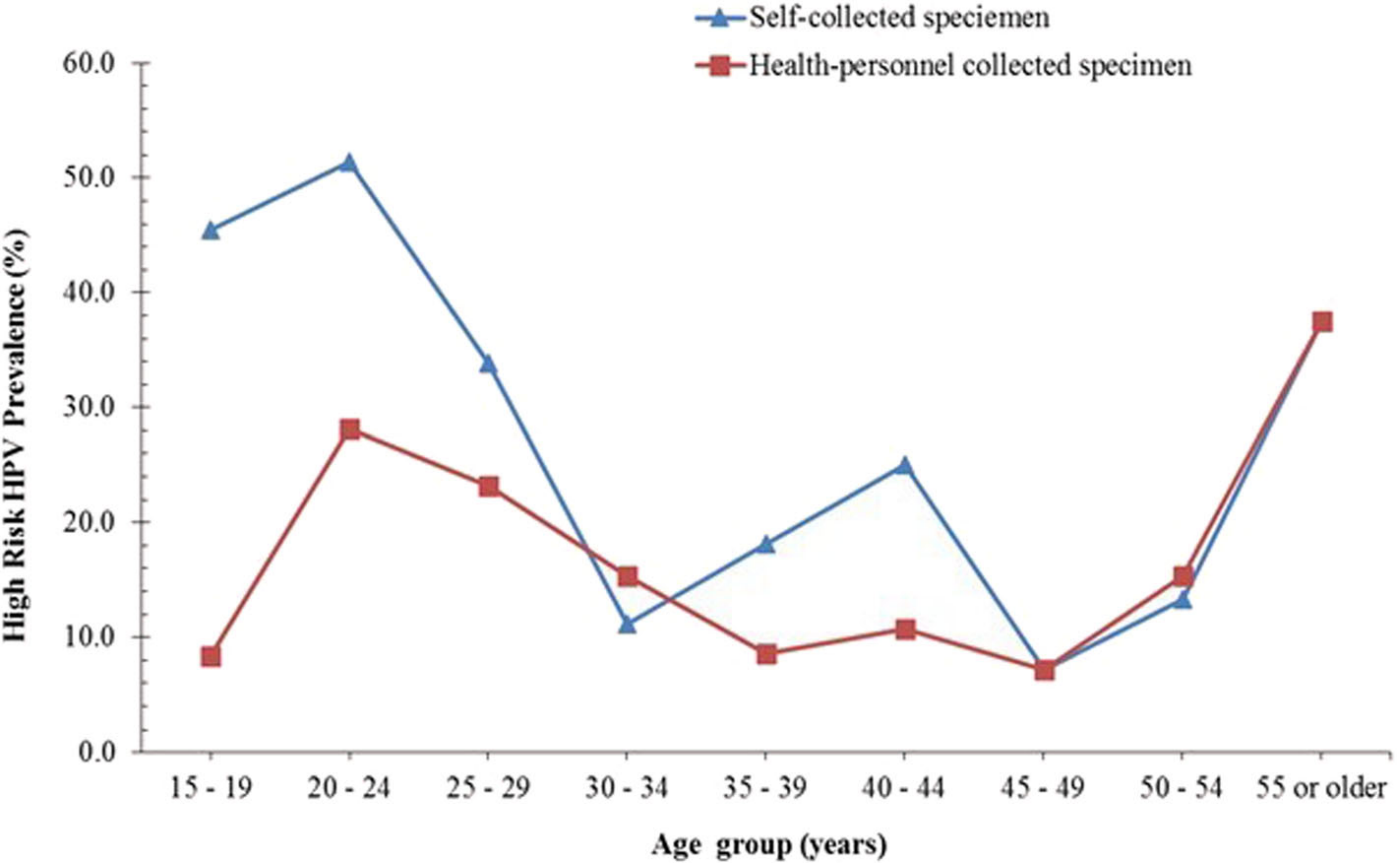
High risk (HR) HPV prevalence curves obtained with self and health personnel collected specimen

**Fig. 3:**
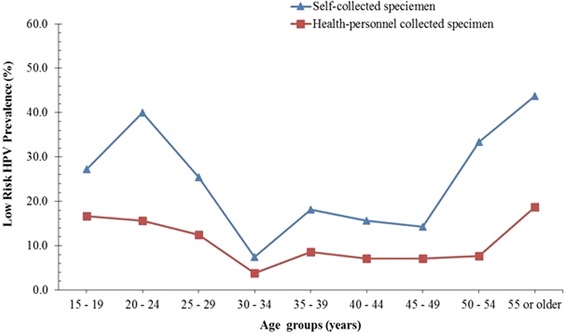
Low risk (LR) HPV prevalence curves obtained with self and health personnel collected specimen

Age was determined to be significantly associated with the following, overall/any HPV positivity (*χ* ^2^ = 36.1; *p* = 0.001), HR HPV positivity (*χ* ^2^ = 26.09; *p* = 0.002) and LR HPV positivity (*χ* ^2^ = 21.49; *p* = 0.011) detected with both self and health personnel collected specimen. The bivariate association controlling for each of the following potential confounders; Age at first sexual intercourse, Lifetime number of sexual partners, Current number of male sexual partners, Contracted a sexually transmitted infection in the past 10 years and Use of condom during sexual intercourses remained significant for each risk type and overall HPV prevalence obtained with self-collected specimen (Additional file 1: Table S1). However, for the health-personnel collected specimen, the association remained significant for overall HPV and HR HPV but become non-significant for LR HPV (Additional file 1: Table S1). With respect to the multivariate association, age remained significant for self-collected specimen determine overall HPV (*χ* ^2^ = 36.44; *p* = 0.0001), HR HPV (*χ* ^2^ = 26.25; *p* = 0.002) and LR HPV (*χ* ^2^ = 20.69; *p* = 0.014). However, for health-personnel collected specimen, the association was significant for both overall HPV prevalence (*χ* ^2^ = 17.76; *p* = 0.038) and HR HPV prevalence (*χ* ^2^ = 17.73; 0.038) but non-significant for LR HPV prevalence (*χ* ^2^ = 8.82; 0.365). The trend of this distribution are as shown in the prevalence curves (Figs. 1, 2 and 3). In respect of the overall HPV infection by both SC and HPC specimen (Fig. 1), a major peak among women of the age group of 20–24 years was followed by a rapid reduction until the age group of 30–34 years. Thereafter, for the SC specimen, a steady rise in prevalence resulted in a second peak at the age group of 40–44 years and sharp drop at age 45–49 years. On the other hand, for the HPC specimen, the prevalence plateaued between the age group 30–34 years and 45–49 years. Then for both SC and HPC specimen, the prevalence increased until among the age group of 55 years or older.

The prevalence curve for high risk HPVs (Fig. 2) was very similar to that of the overall HPV prevalence described above. That is, an initial peak at age group (20–24 years) for both SC and HPC specimen, was followed by a second peak at the age group of 40–44 years for the SC specimen but a plateaued between the age group of 35–39 and 45–49 years for HPC specimen. Interestingly, the subsequent increase in HR HPV prevalence for both the SC and HPC specimen (between 45 and 55 or older years) were almost the same (Fig. 2). Conversely, the low risk (LR) HPV prevalence curve of the SC specimen showed a peak at the age group of 20–24 years followed by a sharp reduction until the age group of 30–34 years. This was followed by a marginal increase at 30 – 34 years and a plateauing until the age group of 45–49 years. The prevalence then increased until the age group 55 or older years. On the other hand, the LR HPV prevalence curve determined with the HPC specimen showed a gradual decreased from a high prevalence among the age of 15–19 years until the age of 30–34 years and then plateaued until the age 50–54 years, then an increase in prevalence was observed at the age group of 55 or older years (Fig. 3).

**Fig. 4:**
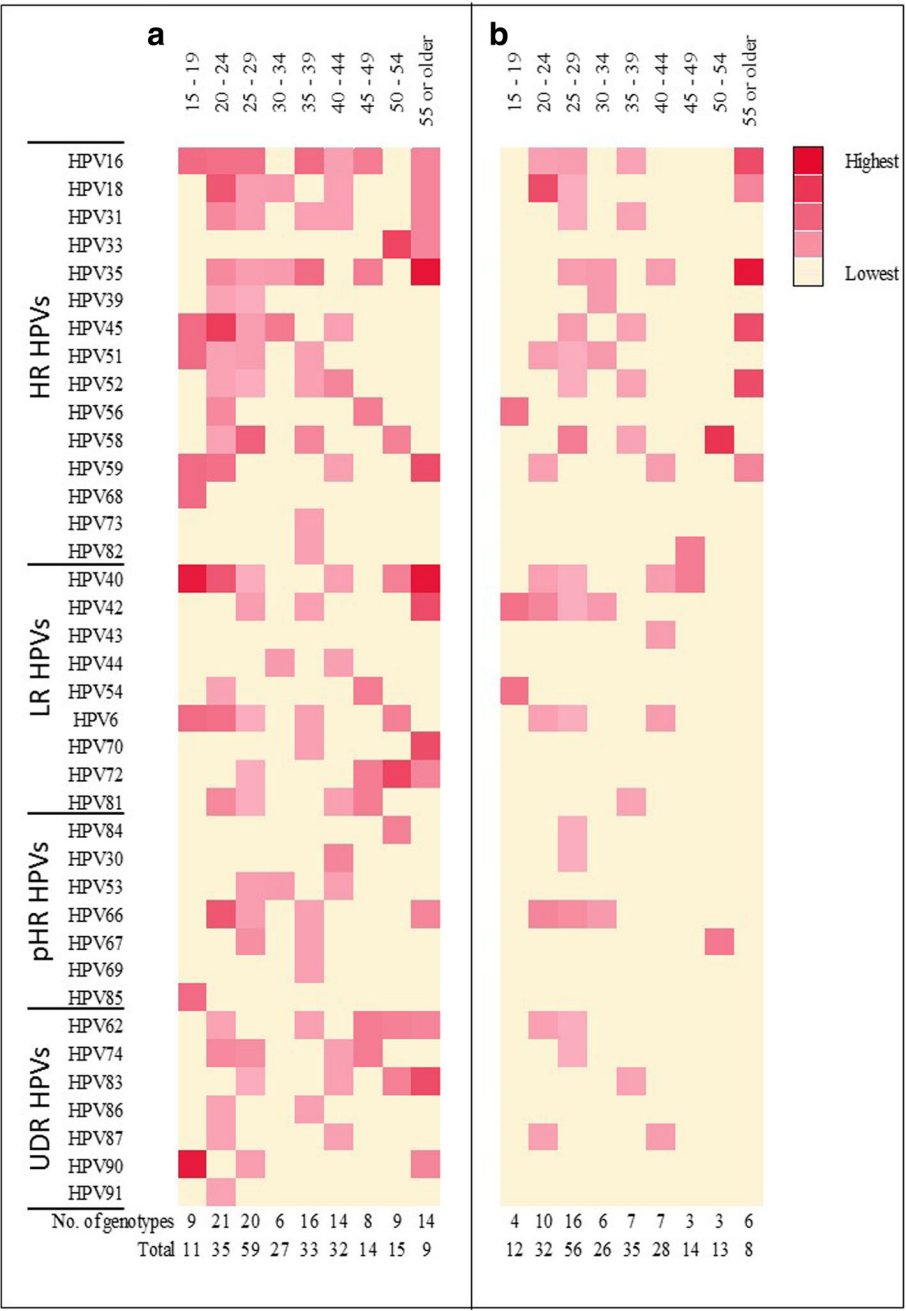
Heat map of the genotype specific prevalence as determined with **a** self-collected specimen and **b** health personnel-collected specimen. HR = high risk; LR = low risk; pHR = probable high risk; UDR = undetermined risk. No. = Number

The age-specific HPV genotype-specific prevalence for each of the detected HPVs are shown as a heat-map (Fig. 4). Clear differences in pattern were obtained with SC specimen as compared to the HPC specimen. A close look at the heat-map for SC specimen showed that the a few HPV genotypes contributed to the mid-age peak (40–45 years) in HPV prevalence curves (Figs. 1, 2 and 3). These were for high risk genotypes, HPV16, 18, 31, 45, 52 and 59; for probable high risk HPV genotypes, HPV 30 and 53 and for low risk HPV genotypes HPV 40, 41 and 81.

### Age-specific concordance between SC and HPC HPV positivity

The concordance between SC and HPC in respect of the *overall HPV positivity* ranged between 53.1 and 60.0% among the aged 15–29 years (Table 3). This range increased among the women in their thirties to between 75.0 and 76.9%. The concordance reduced to 60.7% for women in the age group 40–44 years. Among women 45 years or older, the concordance ranged between 81.3 and 85.7% (Table 3).

**Table 3:** Age-specific concordance of HPV positivity determined with SC and HPC specimen

Grouped age	Both specimens positive for								
	Overall/Any HPV Type			Low Risk HPV Types			High Risk HPV Types		
	Concordance	95% CI		Concordance	95% CI		Concordance	95% CI	
15–19	54.5	48.69	97.43	81.8	52.30	94.86	63.6	35.38	84.83
20–24	53.1	48.41	82.79	75.0	57.89	86.75	62.5	45.26	77.07
25–29	60.0	46.81	71.88	71.4	58.53	81.58	76.8	64.23	85.90
30–34	76.9	57.95	88.97	88.5	71.03	96.00	88.5	71.03	96.00
35–39	75.0	57.89	86.75	90.6	75.78	96.76	84.4	68.25	93.14
40–44	60.7	24.1	76.43	82.1	64.41	92.12	78.6	60.46	89.79
45–49	85.7	60.06	95.99	92.9	68.53	98.73	85.7	60.06	95.99
50–54	83.3	55.20	95.30	66.7	39.06	86.19	91.7	64.61	98.51
55 or older	81.3	47.6	93.2	74.1	44.80	95.14	75.0	64.57	89.24
All ages	67.3	60.89	73.04	79.7	74.03	84.45	78.0	72.14	82.87

Similarly, the concordance between the two methods in respect of the detection of HR HPVs ranged between 62.5 and 63.6% for women aged 15–24 years. However, it increased to 76.8% among the women aged 25–29 years and then even higher among women in their 30s, ranging between 84.4 and 88.5%. It then reduced to 78.6% among women 40–44 years and increased to 85.7% among women 45–49 years and 91.7% among women 50–54 years. Among women 55 years or older, the concordance for HR HPV was 75.0%. However, the concordance between the two methods in respect of LR HPV positivity remained consistently high with age except among women 50–54 years old (Table 3). Despite these variation, the observed differences within the concordance for overall, high risk and low risk HPV detection were not significant.

## Discussion

Overall, our study adds to the evidence of bimodal and trimodal trends in the age-specific HPV prevalence in West Africa and how different this is to those reported for other world regions and countries. For example, a unimodal prevalence curve (20–25 years) in South African [14], Denmark, Spain, Italy, Canada and the Netherlands [12, 31–33]; a bimodal prevalence curve in Hong Kong (26–30 years and 46–50 years) [34], Chine (<25 years and 40–44 years) [35] and in Japan (15–20 years and 50 years) [36]. Additionally, our study provides a more convincing evidence, by using a wider age range, that there was a clear difference in the age-specific HPV prevalences between SC and HPC specimen, which was contrary to those reported by Wright et al., in South Africa [26] and Safaeian et al., in Uganda [27], the only two studies in Africa to have reported similar investigation but within a narrower age range.

The generally higher HR HPV prevalence with SC specimen as well as the additional peak (among the age group 40–44 years) of this curve (Fig. 2), strongly suggest that the use of SC specimens for cervical screening in Ghana will result in additional information on the extent of within-country HPV prevalence variation, which is not likely to be obtained with the use of health personnel-collected specimen. Additionally, the occurrence of a major prevalence peak at the age group of 20–24 years, for both SC and HPC on both the overall and HR HPV prevalence curves, suggests that the use of SC specimen for determining the age-group with a high HPV burden will most likely not be different from that of HPC, rather, it is more likely to give a better indication of the HR HPV infection prevalence at each specific age group in the population. By extension, this potential of SC as a better predictor of the age group at higher risk of developing lower genital tract lesions in future needs to be confirmed by a fellow-up (Figs. 1 and 2).

Comparing the prevalence curves, it is also clear that the mid-age (40–44 years) peak observed in the overall HPV curve for SC was due to the high prevalence of HR HPV for SC among the mid-age group, but not the prevalence of LR HPV. However, other studies have indicated that a higher overall HPV prevalence obtained with SC specimen are often due to a higher LR HPV detection [22, 25]. These studies explained that HR HPV preferentially infects the cervix than the vagina, therefore it was expected that an effectively collected SC specimen should record HPV prevalence at best, similar to that with HPC specimen, but not consistently higher as obtained in our study. Therefore, what remains unclear, in light of the facts that the cytology analysis of the HPC specimen indicated it was well performed (data not shown), is, do these imply that for this population/cohort the vagina harbours more HR HPV infections than the cervix? Furthermore, does the HR HPV prevalence peak at mid-age observed for the SC specimen identify a high risk of cervical lesion that may be missed by using HPC specimen? It is however not clear from these data, whether these additional HR HPV infections detected with SC are infections that may be transient or persist and whether they may lead to the development of lesions. However, the age group showing this additional HR HPV peak prevalence is the age group (30–49) recommended for primary HPV testing, based on the thoughts that this age group mostly harbour persistent HPV infection [37–40].

Comparatively, the middle-age peak of overall HPV and HR HPV prevalences has been shown in some cohort studies with the suggestion that in addition to the persistence of previously acquired HPV infection, the acquisition of new HPV infection by these women accounted for such peaks in prevalence [41, 42]. However in this study, the persistence of the HPV infection were not determined. The only indication that suggest a likelihood of a higher prevalence among this age group is the data on the age at first sexual intercourse, which shows that 70.0% of women within this age group (40–44 years) reported a an AFSI of less than 20 years, which was higher compared to those of the flanking age groups, 25–39 years and 45–59 years (Table 2).

Generally, the high prevalence among younger women (particularly those aged between 20 and 29 years) as has been widely accepted, is related to sexual behaviour, particular those associated with the initiation (AFSI) and conducts of sexual activities [14, 16, 34, 36, 42, 43]. This association was evident in this study since slightly more than 90.0% of the women within the age groups of 20–24 and 25–29 years had at least one current male sexual partner and that only women in the age group of 25–29 years reported 2 and 3 current male sexual partners (Table 2). Also, between 61.5 and 71.4% of the women between 15 and 29 years had contracted an STI in the past 5–10 years prior to the study, compared to the lower proportion in the older age groups. Furthermore, 100 and 87.5% of the women within the age groups of 15–19 years and 20–24 years respectively reported an age at first sexual intercourse of less than 20 years (which is a very significant risk factor for HPV acquisition), while most of the older age groups reported an age at first sexual intercourse of 20 years or more (Table 2).

The high HR HPV prevalence peak among women aged 55 years or older determined with both SC and HPC specimen has been shown only in selected geographical regions. For instance, a very similar age-specific overall HPV and HR HPV prevalence distribution has been reported in a community based study in China, where an initial major peak was observed among women aged 20–24 years, small increase among women aged 34–39 years, a second major peak among women aged 50–54 years [44]. A study in Nigeria showed a first peak and a second high prevalence among women aged between 20 and 24 years and 55–64 years respectively [16, 42]. Another study in Nigeria showed two peaks for women 24–29 years and 55–64 years [16, 42]. Similar high prevalence peak among older women (50 years or older) have also been shown by a meta-analysis for the Central America and West Africa WHO regions [43]. Although the high prevalence among the older women is still not clearly understood, in the larger Nigerian study, no risk factor was identified to have had any significant association (by a logistic regression analysis) with the high prevalence among the age group of 55–64 years [16]. Some studies have suggested that this could have been due to changes in sexual behaviour of both the women and their male sexual partners at that age while other studies have stated that this could be a population specific cohort effect [4, 14, 16, 42, 43]. These population specific cohort effect may include, the lack, low coverage or poor follow-ups of screening and treatment, and some unidentified population characteristic relating to sexual and reproductive behaviour in one population compared to another. For instance, it is common some populations in Ghana for older women to insert all kind of herbal preparations into their vagina with the intension of restoring its elasticity or improving the lubrication thereof. This high prevalence may also result from an increased HPV infection due to the reduced protection (local immunity) and increased dryness of the female reproductive organ as a result of menopausal changes [45, 46]. For instance, during or after menopause, there is a reduction in the secretions of the female reproductive organ, which are known to protect against infections and provide lubrication that reduce/prevent the occurrence micro-abrasion and subsequent HPV infection [46–48]. However, it was not possible in this study to comment extensively on these due to the small number of participants in sub-group analysis. However, for a population with a relatively lower life-expectance (<80 years), and given the natural history of HPV and cervical cancer, which indicates that a period of 10–15 years passes before a few of the HR HPV infection may progress to cervical lesion [10], most of the women may not be alive to develop the disease and therefore HPV based screening among women of this age group may have very little value, if any, particularly in developing countries.

It was important to determine whether the observed difference between SC and HPC specimen prevalence curves, for both the overall and HR HPV prevalence curves, (Figs. 1 and 2) resulted in significant difference in the age-specific concordance between the two collection methods. This will show if the overall concordance between SC and HPC vary significantly with age and if for all ages SC specimen may be used in place of HPC specimen. Considering the age-specific overall HPV prevalence concordances, it was observed that for the age group that SC overall HPV prevalence was much higher than that of HPC (15–19 years and 20–24 years), the concordance were lower (Fig. 1 and Table 3), and where the difference between their prevalence was lowest (30–34 years; 45–49, 50–54 and ≥ 55 years), the concordances were highest. These were also evident with the HR HPV and LR HPV prevalence curves and concordances. Do these suggest that the variation in the concordance between SC and HPC reported by most studies may be related to the HPV types (LR HPV) that preferential infection of the vagina and possibly more among younger women as well as with how high the overall HPV prevalence is for that study? That is, for a population with a high HPV prevalence, there is a high likelihood of a lower concordance between SC and HPC HPV prevalence and vise versa. A close look at the mini review by Gravitt et al. shows a similar trend between the SC HR HPV prevalence and the measure of agreement (Kappa values) between SC and HPC methods [22]. Furthermore, it was interesting to note that the highest concordances in our study were among women between 30 years and 54 years, an age range recommended for HPV testing (in co-testing) [38]. Therefore, the use of SC based HPV testing instead of HPC based HPV testing is further strengthened, since the best concordances were within these age group that need to be screened for persistent HPV.

The results and subsequent conclusions of our study must be viewed in the light of the fact that the number of participants was not even distributed the across age groups and was limited in some of the older age groups, as such resulted in wider 95% confidence intervals and in some cases did not allow for the further more rigours statistical analyses.

## Conclusions

Based on these findings, the existence of a bimodal and or a trimodal HPV prevalence in some Ghanaian population is to be expected in future investigations and also that the use of SC specimen is mostly likely going to provide more information in respect of the HR HPV burden. In respect of the difference between age-specific HR HPV prevalence curve, and concordance between SC and HPC, further investigations are needed to throw more light on the question, does the overall HPV prevalence (particularly determined with SC) and/or age of women influence the level of concordance between HR HPV test by SC and HPC specimen? Further investigation will shed light on the possible utility of HR HPV testing with SC or HPC in determining screening recommendations for this and other similar populations in low income countries..

## Additional file

Additional file 1: Table S1. Bivariate and multivariate Chi-square association of age and HPV infection. (DOCX 12 kb)

## Abbreviations

CI: Confidence interval
EDTA: Ethylene diamine tetraacetic acid
ERC: Ethical Review Committee
GHS: Ghana Health Service
HPC: Health personnel collected
HPV: Human Papillomavirus
OR: Odds ratio
PE: Streptavidin-phycoerythrin
SC: Self-collected
TMAC: 1-tetramethyl ammonium chloride
