# Supplementary material for: Differences in age-specific HPV prevalence between self-collected and health personnel collected specimen in a cross-sectional study in Ghana
Source: Infect Agent Cancer. 2017 May 18;12:26. doi: 10.1186/s13027-017-0136-7 (PMC5437497; doi:10.1186/s13027-017-0136-7)
Supplement: Additional file 1: Table S1. — Bivariate and multivariate Chi-square association of age and HPV infection. (DOCX 12 kb) [file 13027_2017_136_MOESM1_ESM.docx]

**Table S1: Bivariate and multivariate Chi-square association of age and HPV infection**

| **Risk factors adjusted for** | **Self-collected specimen, χ^2^ (p value)** | | | **Health personnel collected specimen, χ^2^ (p value)** | | |
| --- | --- | --- | --- | --- | --- | --- |
|  | **Overall HPV** | **HR HPV** | **LR HPV** | **Overall HPV** | **HR HPV** | **LR HPV** |
| Lifetime number of sexual partners | 35.89 (0.001) | 25.79 (0.002) | 21.29 (0.011) | 18.46 (0.030) | 18.93 (0.026) | 10.39 (0.319) |
| current number of sexual partners | 35.19 (0.001) | 26.09 (0.002) | 21.49 (0.011) | 18.70 (0.028) | 19.16 (0.024) | 10.47 (0.313) |
| Sexual age | 37.72 (0.001) | 27.27 (0.001) | 21.45 (0.011) | 18.49 (0.030) | 18.33 (0.032) | 10.11 (0.342) |
| STI in the past 10 years | 35.25 (0.001) | 25.39 (0.003) | 20.94 (0.013) | 18.19 (0.033) | 18.81 (0.027) | 10.25 (0.330) |
| Use of condom | 35.89 (0.0001) | 25.79 (0.002) | 21.29 (0.011) | 18.46 (0.030) | 19.93 (0.026) | 10.39 (0.319) |
| Age at first sexual intercourse | 37.72 (0.0001) | 27.27 (0.0001) | 21.45 (0.011) | 18.49 (0.030) | 18.38 (0.032) | 10.11 (0.342) |
| All factors | 36.44 (0.0001) | 26.25 (0.002) | 20.68 (0.014) | 17.76 (0.038 | 17.73 (0.038) | 8.82 (0.365) |
